# Supplementary material for: Brainstem networks construct threat probability and prediction error from neuronal building blocks
Source: Nat Commun. 2022 Oct 19;13:6192. doi: 10.1038/s41467-022-34021-1 (PMC9582012; doi:10.1038/s41467-022-34021-1)
Supplement: Supplementary file 1 — Supplementary Information [file 41467_2022_34021_MOESM1_ESM.pdf]

Brainstem networks construct threat probability and prediction error from neuronal building blocks

Jasmin A. Strickland[1,2]* and Michael A. McDannald[1]*

[1]Boston College, Department of Psychology & Neuroscience, Chestnut Hill, MA, 02467, USA
[2]Department of Psychology, Durham University, DH1 3LE, UK
*Email: jasmin.strickland@bc.edu and michael.mcdannald@bc.edu

Supplementary Methods

Head cap preparation

Head cap designs were created using FreeCAD and Tinkercad then 3D printed using a Form2 printer with clear resin (RS-F2-GPCL-04). Prints were washed and rinsed with 90% ethanol before drying for at least 48hrs. The head cap consists of a base section, lid, and probe mount. The probe mount slots into the front section of the cap and the head stage fits into the back section. Neuropixels probes were glued to the probe mount using a small amount of superglue on the back of the probe base. Stainless steel ground wire was soldered to the ground and reference pads located at the top of the wings of the probe. The head stage was attached to the flex cable before the probe mount/probe was carefully inserted into the front section of the head cap, with the ground wire inserted through a small hole in the bottom of the cap. The head stage sits in the back part of the cap with the flex cable forming an S-shape inside the cap (supplementary Fig. 1a). The probe mount and head stage are then fixed in place with small dots of dental cement. The lid is secured with fine adjustment nylon cable ties (McMaster Carr: 6614K11). A separate lid was designed and printed for recording sessions and attached to the end of the plastic cable shielding. The recording cable could then run through the shielding and into the head cap. The recording head

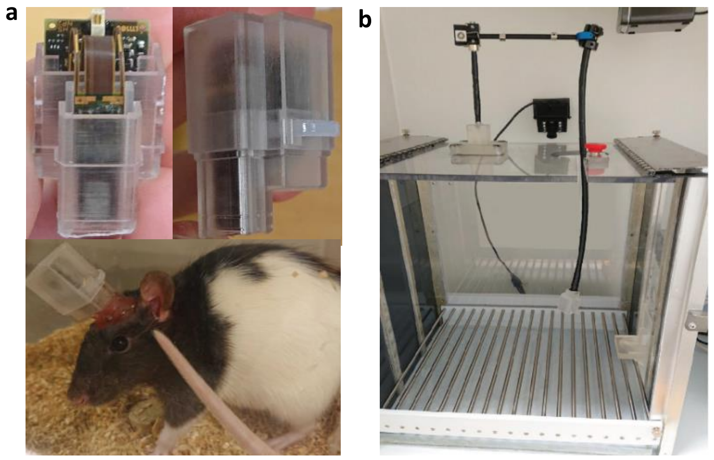

Supplementary Fig. 1 **Neuropixels headcap and setup**. (a) Images of Neuropixels headstage and probe, in preparation and head fixed to a rat. (b) Multi-axis arm, cable, and headcap lid for recording.

cap was held by a multi-axis rotating arm (Instech: MLCA) to allow free the rat free movement during recording sessions (supplementary Fig. 1b).

Surgery

Prior to surgery vacuum grease was applied around the base of the front part of the head cap avoiding any grease touching the probe. The probe was then carefully painted with DiI (ThermoFisher, V22885). The skull was leveled, dried, and scored in a crosshatch pattern before four screws were fixed into the skull at locations leaving sufficient space for the head cap. A 1.4mm craniotomy was carried out and the dura removed. A clamp (southern labware: 30392204) was modified so that it could be used as a stereotaxic arm and hold the head cap. A piece of rubber tubing was put around the head cap to give the claw a better grip. The clamp holding the head cap was fixed to the stereotaxic arm at a 15º angle, centered over the craniotomy, and slowly lowered until the probe came into contact with the cortex. The flat edge of a needle was carefully placed alongside the probe to prevent excessive bending and to help pierce the cortex. Once the probe visibly entered the cortex, it was slowly lowered until about half of the implant final depth was reached (~3.5mm). Using a syringe and needle a small amount of the silicone compound (Dow: DOWSIL 3-4680) was injected into the craniotomy around the probe and given at least 5 minutes to set. The head cap was then lowered to its final position, either until the max depth was reached (7.5mm) or the head cap base came into contact with the skull. Additional grease was applied around the base of the cap, sealing any gaps between the cap and the skull. The ground wire was wrapped around the two nearest skull screws in a figure of 8 pattern to ground the probe. The head cap was then cemented in place by filling the surrounding area with orthodontic resin (cc 22-05-98, Pearson Dental Supply), with care taken to ensure the cement reaches underneath the upper part of the head cap and around the entire base. A final amount of silicone gel was injected into the front part of the head cap to ensure the craniotomy was sealed. Once the cement and gel was set the clamp was released from around the head cap and the lid fixed in place with a cable tie.

Single unit data acquisition

An acquisition computer was connected to a PXIe-1071 chassis containing an acquisition module, a remote control module (PXIe-8381), and an I/O module (PXI-6363) with attached BNC connector block (2110). A med associates operant chamber was controlled using a separate computer running med-associates software. Behaviour data was collected using med-associates TTL boxes (SG-231) attached to the connector block. Single unit data was acquired

using OpenEphys software with the Neuropix-PXI module[1]. The NI-DAQmx module[2] was used to record analogue signals of different lengths to timestamp events and behaviour of interest.

During each recording session the head cap lid was removed by cutting away the cable tie. The Neuropixels cable coming through the recording head cap was attached to the head stage and the recording lid secured with a new cable tie. The signal from the probe was checked in OpenEphys, and if successfully acquired without excessive noise or breaks in signal the recording channels for that session were selected in the Neuropix-PXI module. External or internal reference was selected to maximize the signal to noise ratio during recording. In the NI-DAQmx module all digital channels were unselected and the 3 analog channels for collecting behaviour data selected. The behaviour program was then started alongside recording in OpenEphys. Due to the large file size data were recorded directly to an external hard drive.

If signal was not acquired on first plugin in attempt the rat was re-plugged in. If signal was not able to be acquired after repeated plugin attempts signal was considered lost and recording session not carried out. If signal was still no longer able to be acquired the following day signal was considered lost and recording sessions ended for that subject. If signal was lost during the first ~10 minutes of a session, the session was ended and re-started if signal was able to be re-acquired. If signal was lost further into the session, then the session was lost and recording ended for that subject for that day and resumed the following day. A camera inside the chamber allowed continued monitoring of the rat during the recording session. At the end of the recording session the rat was removed from the chamber and the lid but back onto the head cap with a new cable tie.

Probe explant

After recording sessions were completed or terminated, the rat was put back under isoflurane anesthesia and returned to the stereotax in order to secure the head. The lid was removed, and the ground wire cut inside the head cap. Using a scalpel, the small dots of cement keeping the head stage in place were scraped off and the head stage removed. Next, the cement securing the probe mount was scraped off, and the mount very slowly pulled up/out of the head cap. Special care was taken not to pull too hard, otherwise the silicon in the bottom of the head cap may cause the probe to break. The retrieved probe was briefly soaked in DI water before being soaked overnight in a 1% tergazyme solution to remove residue silicon and biological material. Probes were then given a final rinse with DI water and 90% IPA before stored for future use.

Identifying probe tracts

Immediately following probe retrieval rats were deeply anesthetized perfused intracardially and brains extracted. Extracted brains were frozen then sectioned for the full anterior posterior extent of the probe. Sections were mounted and processed with NeuroTrace, a cell body marker. Images of the full probe path were acquired with an Axioimager Z2 microscope. X/Y/Z coordinates were determined for the tip (most dorsal location) and top (most ventral location) of each probe implant. An implant vector was calculated based on these coordinates and each micrometer along the probe assigned x/y/z brain coordinates.

Data processing and single unit sorting

All unit sorting and data analysis were carried on an analysis computer with a GeForce GTX 1080 GPU and 64GB RAM. The aim of the unit sorting process was to ensure that each single unit accepted for analysis met the criteria of being an isolated single unit and was held for the entire 16 trials of the session in which it was recorded. Units were sorted using Kilosort2 or Kilosort2.5. Sorted sessions were loaded into Phy for manual curation process. Each unit identified by Kilosort was individually assessed in Phy using the ISI, correlogram, waveform, and feature view, and compared against other units for splits and merges. The temporal view was also examined to see if activity was recorded throughout the session or was dropped partway through. If a unit did not show clear separation from noise and/or other units, and/or did not show expected patterns of single unit activity (e.g. clear refractory period) it was not labelled as good and excluded from further analysis. On average 45.9 units per recording session were labelled as ‘good’ in phy and accepted for further analysis.

Using Matlab (with code from the Cortex-lab spikes repository[3] information about firing and single unit location along the probe was extracted from the phy output for each session. Behaviour timestamps (danger/uncertainty/safety cues on, shock on, nose poke, pellet reward delivery) were extracted from the 3 analogue channels using a Matlab. A subsequent Matlab script created a single .mat file for each single unit that contained waveform time stamps, behaviour timestamps, and location on probe. Single units were then screened for stability across the recording session. Any unit that showed clear loss of activity during any of the trials was excluded from the analysis. Of the units that were still included at this stage, 52.6% met criteria to be included in the final analysis. During the screening stage it became apparent that there was a drift in the behaviour/events timestamps relative to the single unit firing data across each recording session. Using the timestamps recorded on the behaviour acquisition computer to compare to the timestamps extracted from the analogue data streams feeding into OpenEphys the drift was calculated and a correction applied to each single unit accepted for

analysis (0.26s drift in the behaviour time stamps occurred during the 54-minute recording session). Next the coordinates on the probe that each single unit was recorded from were extracted from the single unit Matlab files. For each subject the location that each single unit was recorded on the probe was mapped onto the coordinates calculated for that implant. These coordinates were then overlaid onto the Paxinos and Watson rat atlas[4] in Matlab. Using these overlays and assigned coordinates each unit could then be manually assigned to a specific brain region. ‘Units’ obtained from the central aqueduct were removed. The final stage of unit processing was to run a cube script that compiled all the 1812 single unit Matlab files into a single array for analysis in Matlab.

Z-score normalization

For each single unit and trial, firing rate (Hz) was calculated in 250 ms bins from 20 s prior to cue onset to 20 s following cue offset, for a total of 240 bins. Differential firing was calculated for each of the 240 bins by subtracting baseline firing rate (2 s prior to cue onset) specific to that trial type from each bin. Differential firing was *Z*-score normalized across all trials for each single unit, such that mean firing = 0, and standard deviation in firing = 1. *Z*-score normalization was applied to firing across the entirety of the recording epoch, as opposed to only the baseline period, in case single units showed little/no baseline activity. As a result, periods of marked firing increase during cue presentation would contribute to normalized mean firing rate (0). For this reason, *Z*-score normalized baseline activity can differ from zero.

Heat plots

Heat plots were constructed from normalized firing rate using the imagesc function in Matlab. Mean normalized firing rate was calculated for each trial type for each single unit. For the cue heat map, this meant three trial types: danger, uncertainty, and safety. For the outcome heat map, this meant four trial types: danger, uncertainty shock, uncertainty omission, and safety. Perceptually uniform color maps were used to prevent visual distortion of the data[5].

K-means clustering

For the cue period, brainstem single unit firing was organized into an 1812 single unit x 42-time interval matrix containing 14 x 1s intervals for each cue (1-14 danger, 15-28 uncertainty, 29-42 safety). These 14 intervals included the 2s pre-cue,10s cue, and 2s following cue offset but before shock delivery. Clustering was carried out using the Matlab kmeans function. K-means clustering results were number identifier for each single unit and the mean of the squared

Euclidean distance from cluster centroid. K-means clustering was performed 25 times, incrementing the number of clusters from 1-25. For each iteration we compared the number of clusters, the mean of the squared Euclidian distance and the number of single units composing the clusters. These three factors were examined for over and under clustering. Over clustering was apparent when clusters contained only a single or low number of single units, and when adding additional clusters marginally reduced the mean squared Euclidean distance. Under clustering was apparent when clusters had large mean squared Euclidean distances and dissimilar single unit types were lumped together. The ideal # of clusters for each firing period found the midpoint of these extremes. K-means clustering was performed identically for the outcome period, only now firing data came from the post shock period. An 1812 single unit x 80 time-interval matrix was constructed from the 20, 500ms intervals for each trial type 10s from shock offset (1-20 danger, 21-40 uncertainty shock, 41-60 uncertainty shock omission, 61-80 safety). K-means clustering was performed 20 times, incrementing the number of clusters from 1-20.

Principal components analysis

Principal component analysis (PCA) was performed to reveal low-dimensional brainstem firing features. Brainstem single-unit firing was organized into an 1812 single unit x 42 column matrix (14, 1s intervals for each trial type) for the cue period; and an 1812 x 80 column matrix (20, 500ms intervals for each trial) matrix for the outcome period. PCA was performed using the PCA function in Matlab. PCA outputs were the PC weights (cue x time) for each principal component as well as the percent firing variance explained for each principal component. Cluster identity from k-means clustering, principal components analysis combined with iterative shuffling to determine each clusters contribution to principal component 1. First, the percent firing variance explained by PC1 was determined for all brainstem single units as described above. This result served as the ground truth reference for subsequent results. Next, a single cluster was selected (i.e., cluster 21). Firing information for the single units of only that cluster were shuffled. Firing information for all other clusters (i.e., clusters 1-20) was left intact. Principal components analysis was performed on this modified data set (clusters 1-20 intact, cluster 21 shuffled) and the percent firing variance explained for PC1 determined. PC1 contribution for that iteration was determined by subtracting the modified data set (clusters 1-20 intact, cluster 21 shuffled) from the ground truth (all clusters intact). Shuffling → PCA was performed 1,000 times for each cluster, and PC1 contribution determined for each iteration, then averaged. The complete iterative, shuffle/PCA was performed for each individual cluster, always shuffling the

cluster of interest but leaving intact all other clusters. Clusters more greatly contributing to PC1 would have larger difference values, indicating that removing their firing information more greatly reduced the percent of firing variance explained by PC1.

Danger firing latency

Mean normalized firing rate during 10-s danger cue presentation was summarized in 10,000, 1ms bins for each single unit. Danger firing latency was determined for each single unit by identifying the bin of greatest firing inflection (among the 10,000 total during danger presentation) using the findchangepts Matlab function. Mean danger firing latencies were calculated for each cluster by averaging the latencies of the composing single units. Between-cluster differences in latency means were determined using Bonferroni-corrected (p = 0.05/# of tests), independent samples t-tests. Between-cluster differences in latency variation were determined using Bonferroni-corrected, Levene's test for equality of variances.

Correlated cluster firing

Mean cue firing for each cluster was organized into an 1812 single unit x 36 column matrix (12, 1s intervals for each cue, 2s pre-cue through 10s cue). Mean cue firing for each cluster was correlated with mean cue firing for every other cluster. The result was a 21 x 21 matrix of the Pearson’s correlation coefficient (R^2) for each cluster comparison. Correlation coefficients on the diagonal were always 1, as they were a cluster’s correlation with itself. Correlated cluster firing was also performed for the outcome period, for which cluster outcome firing was organized into an 1812 single unit x 80 column matrix (20, 500ms intervals for each trial type, 10s following shock offset).

Hub identification with single unit x cluster correlations

For each single unit from each of the eight cue subnetwork clusters (i.e., cluster 1), single-unit firing during the danger cue (14 x 1s intervals, starting 2s pre-cue) was correlated against mean danger firing of each remaining seven clusters (i.e., clusters 2-8). The result was seven separate correlation coefficients for each single unit (R^2), one for each cluster comparison. A mean correlation coefficient was calculated for each single unit, providing a measure of how well each unit correlated with the firing of the other subnetwork clusters. Independent samples t-tests were used to determine differences between-cluster differences in mean R^2. Levene’s test for equality of variance was used to determine differences between-cluster differences in standard deviation of R^2. Bonferroni correction was applied to both comparisons, producing a

significance threshold of 0.007 (0.05/7). A cluster showing the highest mean and least variable R^2 was considered a hub. Similar analyses were performed for the phasic outcome subnetwork (clusters 1-5) and the tonic outcome subnetwork (clusters 7-10); only now, firing was taken from all trials types (20, 500ms intervals for each trial type, 10s following shock offset).

Linear regression

Linear regression was used to determine whether cue cluster firing was better explained by threat probability or behaviour. Separately for each of the 21 cue clusters, firing was organized by trial type (16 trials, 3 types) over 14, 1s intervals (2s pre-cue through 2s post-cue). The threat probability regressor was the foot shock probability associated with each cue (1 or 0.25 or 0). The behaviour regressor was the trial-specific, mean suppression ratio for the cluster. The Matlab regress function was used simultaneously compare which regressor better explained cluster firing for each interval. Regression output was a beta coefficient for each regressor and interval (fig 3A). A beta coefficient quantified the direction (>0 = positive) and strength (>0 = stronger) of the relationship between each regressor and cluster firing. Regression was also performed for the 11 outcome clusters. Firing was organized by trial type (16 trials, 4 types) over 20, 500ms intervals (10s following shock offset). The regressors were shock: 1 (danger), 1 (uncertainty shock), 0 (uncertainty omission), and 0 (safety); and prediction error: 0 (danger), 0.75 (uncertainty shock), -0.25 (uncertainty omission), and 0 (safety). Regression output was a beta coefficient for each regressor and interval. A beta coefficient quantified the direction (>0 = positive) and strength (>0 = stronger) of the relationship between each regressor and cluster firing.

'Lesion' x principal components analysis x linear regression

Principal component analysis (PCA) was performed to reveal low-dimensional brainstem signaling features. Cluster beta coefficients for threat probability and behaviour were organized into a 21-cluster x 28-interval matrix (intervals 1-14 probability, 15-28 behaviour) for the cue period. PCA was performed using the PCA function in Matlab. PCA outputs were the weights (regressor x time) for each principal component (we focus on PC1 and PC2) as well as the percent signaling variance explained by PC1 and PC2 (Fig 2C). PCA was first performed with all clusters and networks 'intact'. PCA was similar for the outcome period, only now cluster beta coefficients for signed prediction error and shock were organized into a 21-cluster x 40-interval matrix (intervals 1-20 shock, 21-40 prediction error) for the post-shock period. The 'lesion' analysis was similar to that used for determining the contribution of each cluster to low

dimensional firing features. Only now, the lesion approach was used to determine differential contributions of the cue subnetwork and supranetwork to low dimensional cue signaling; as well as differential contributions of the phasic and tonic outcome subnetworks to low dimensional outcome signaling.

First, the PC1 and PC2 weight patterns (regressor x time) were determined for all 21 clusters as described above. Next, subnetwork clusters 1-8 were selected. Beta coefficients for only these cluster were shuffled. Principal components analysis was performed on this modified data set (clusters 1-8 shuffled, clusters 9-21 intact) and the PC1 and PC2 weight patterned determined. Shuffling → PCA was performed 1,000 times, and PC1/PC2 weights determined for each iteration, then averaged for all 1,000 iterations. All signaling by the cue subnetwork would be lost, while signaling by the supranetwork would be intact. The inverse analysis was performed to determine signaling by the supranetwork: subnetwork clusters were intact but supranetwork clusters were lesioned. Finally, the outcome 'lesion' analysis contrasted contribution of the phasic and tonic outcome networks by shuffling one network, while leaving the other intact. Comparing PC1 and PC2 weights for the fully intact regression x PCA versus phasic shuffled and tonic shuffled allowed to reveal the specific contribution of each network to outcome signaling.

Mapping cluster and network membership to brain region

For each cue and outcome cluster, the percent of member single units originating from each brain region was determined and plotted using the Matlab bubchart function. To map network membership to brain region, the percent of single units from each region contributing to the subnetwork vs. supranetwork and phasic vs. tonic outcome network were calculated and visualized with donut charts using the donut function in Matlab. To determine the differential contribution of each region to functional networks, first the population proportions of subnetwork vs. supranetwork and phasic vs. tonic outcome network were determined. Then for each brain region, the proportion contributing to each network was subtracted from the overall proportion. Values around zero would indicate a distribution of subnetwork vs. supranetwork and phasic vs. tonic outcome network clusters that reflected the entire brainstem. Deviations from zero would indicate preferential network contributions.

Determining temporal firing pattern following shock

Temporal firing pattern following foot shock was calculated by averaging normalized z firing following shock on danger and uncertainty trials. Differential firing was then calculating mean

normalized firing 1s following shock offset – mean normalized firing 5s following shock offset. The absolute value was taken to fairly compare units increasing vs. decreasing firing following foot shock. Phasic neurons will have high values, while tonic neurons will have low values.

Relationship between cue network and outcome network membership

Chi square testing was used to determine the relationship between cue subnetwork membership and outcome network membership. 505/1812 (27.9%) single units were in the cue subnetwork. Therefore, chi-square test determined if the proportion of phasic outcome single units in the cue subnetwork differed from 27.9%. The same was asked for the tonic outcome single units. Results were a chi-squared statistic and associated p value.

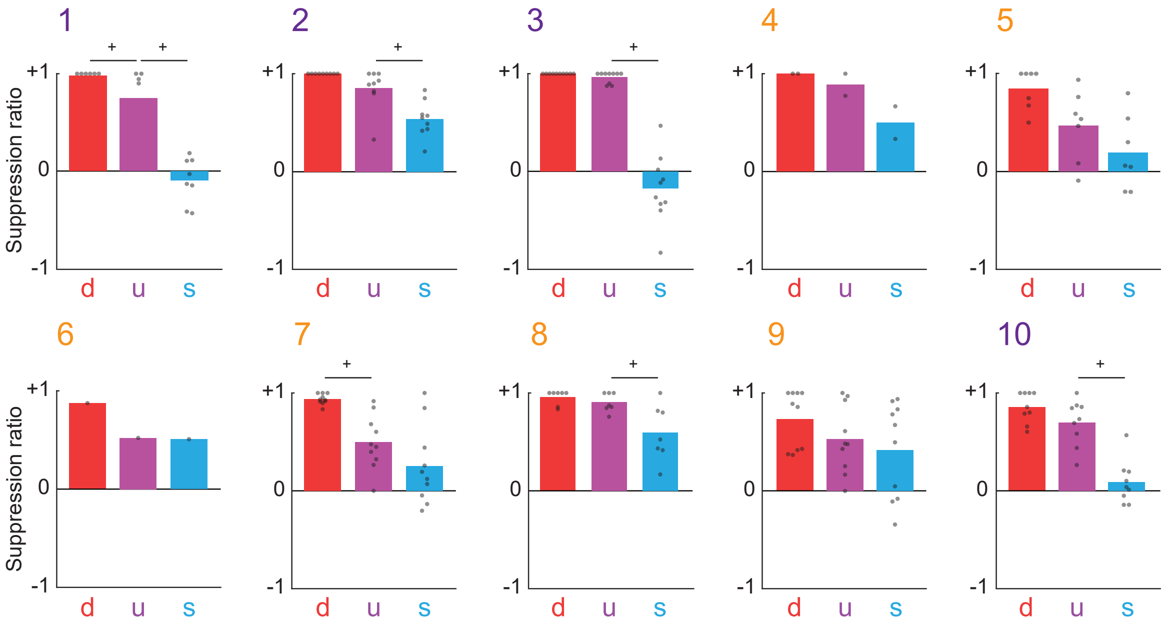

Supplementary Fig. 2 **Suppression ratios for each rat.** Females (1-3, 10), males (4-9).
⁺95% bootstrap confidence intervals do not contain zero. Plot spread n = number of recording sessions.
Abbreviations: d, danger; u, uncertainty; and s, safety

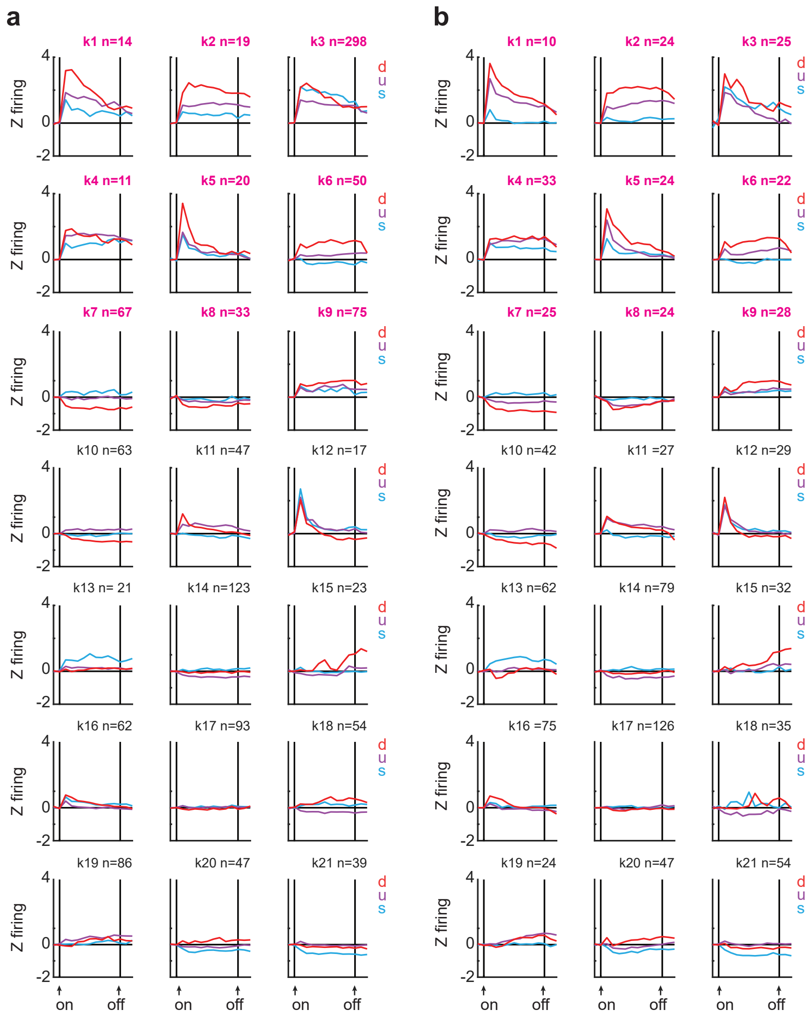

Supplementary Fig. 3 **Brainstem cue cluster firing, split by sex.** Mean cluster (K1-21) firing over cue presentation (cluster n shown in figure), for units recorded from female rats (a) and male rats (b). Abbreviations: d, danger; u, uncertainty; and s, safety.

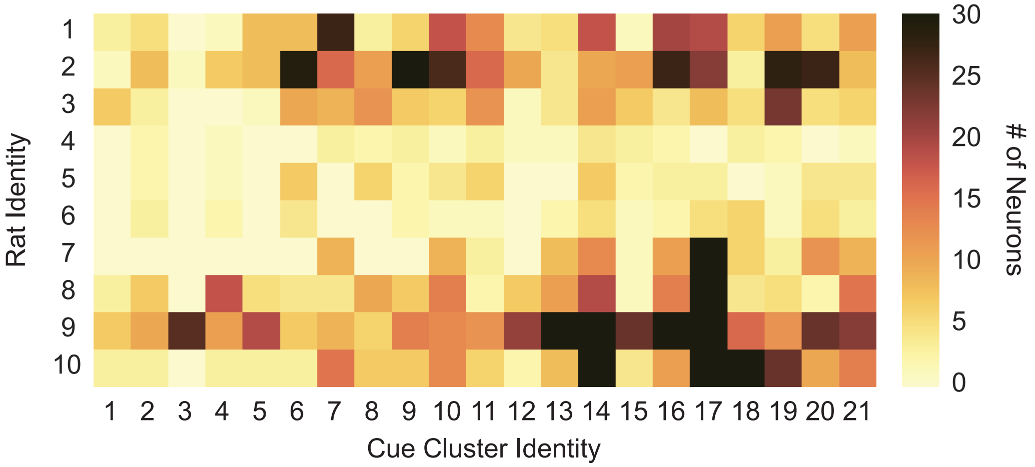

Supplementary Fig. 4 **Rat identity x cue period cluster identity** correlation matrix

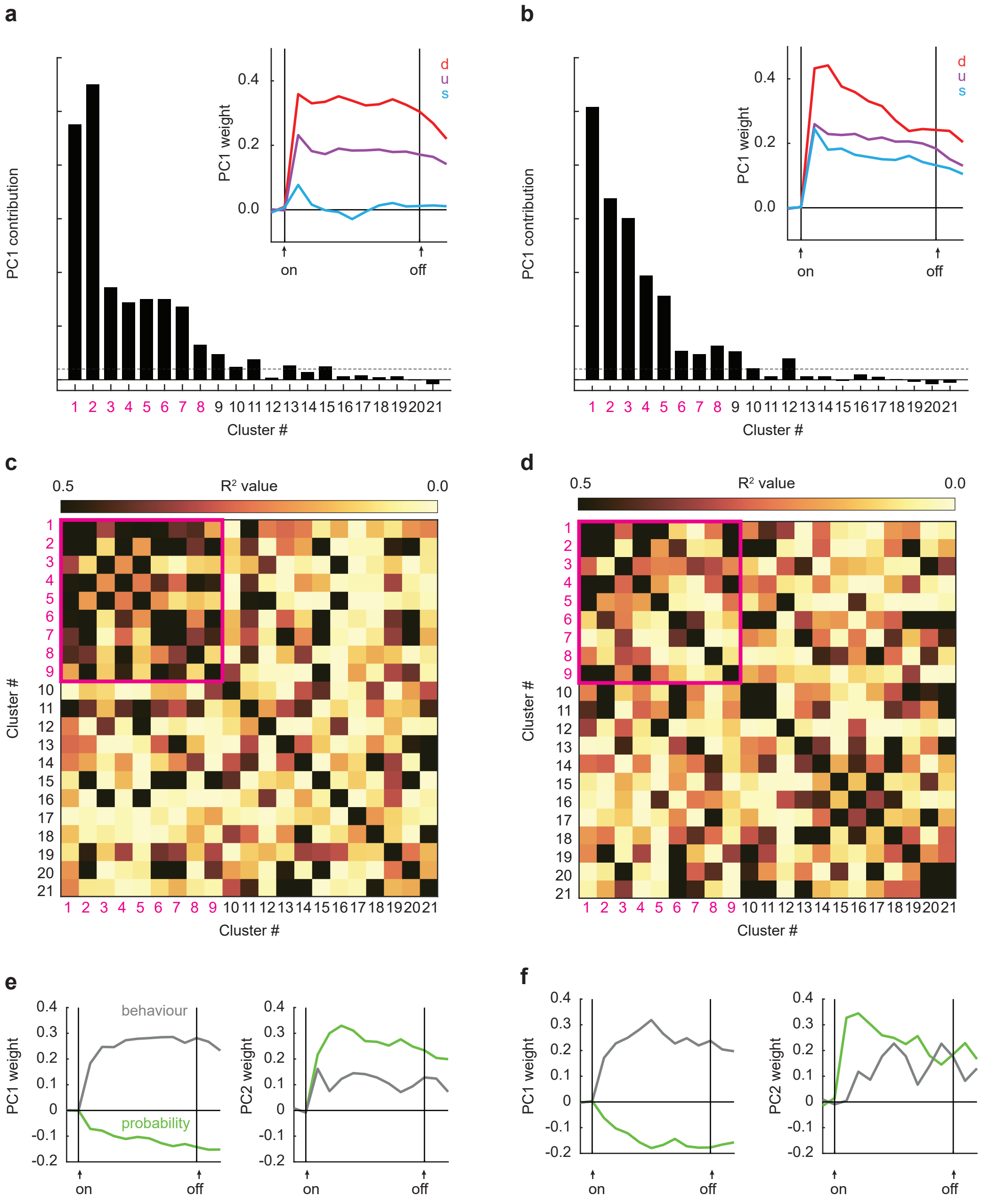

Supplementary Fig. 5 **Brainstem cue firing, split by sex**. PC1 for cue firing (inset) and cluster contribution to PC1 for units from female rats (a) and male rats (b). Between cluster cue-firing correlations for units from female rats (c) and male rats (d), cue subnetwork outlined in magenta. PC1 and PC2 from principal components analysis for cluster beta weights for units from female rats (e) and from male rats (f). Abbreviations: d, danger; u, uncertainty; and s, safety.

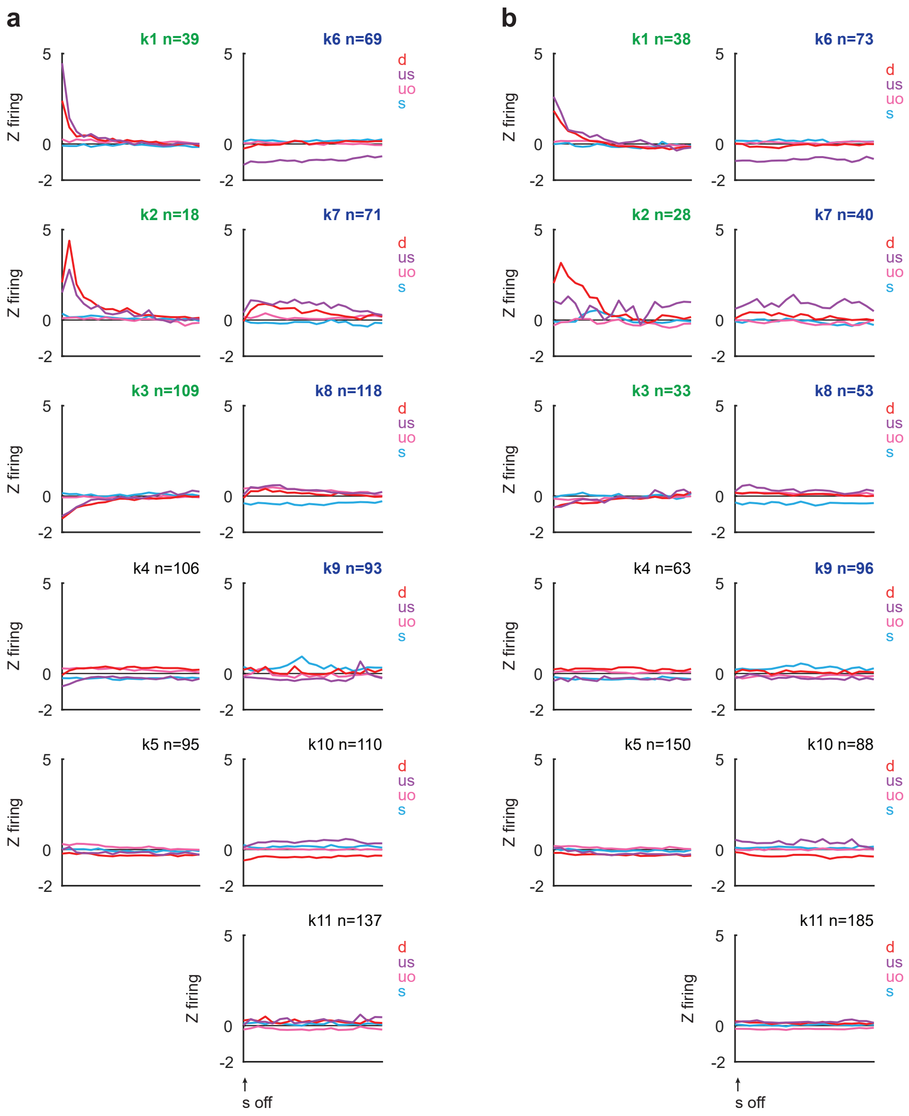

Supplementary Fig. 6 **Brainstem outcome cluster firing, split by sex.** Mean cluster (1-11) firing following shock (cluster n shown in figure). (a) units from female rats, (b) units from male rats. Abbreviations: d, danger; us, uncertainty shock; uo, uncertainty omission, and s, safety.

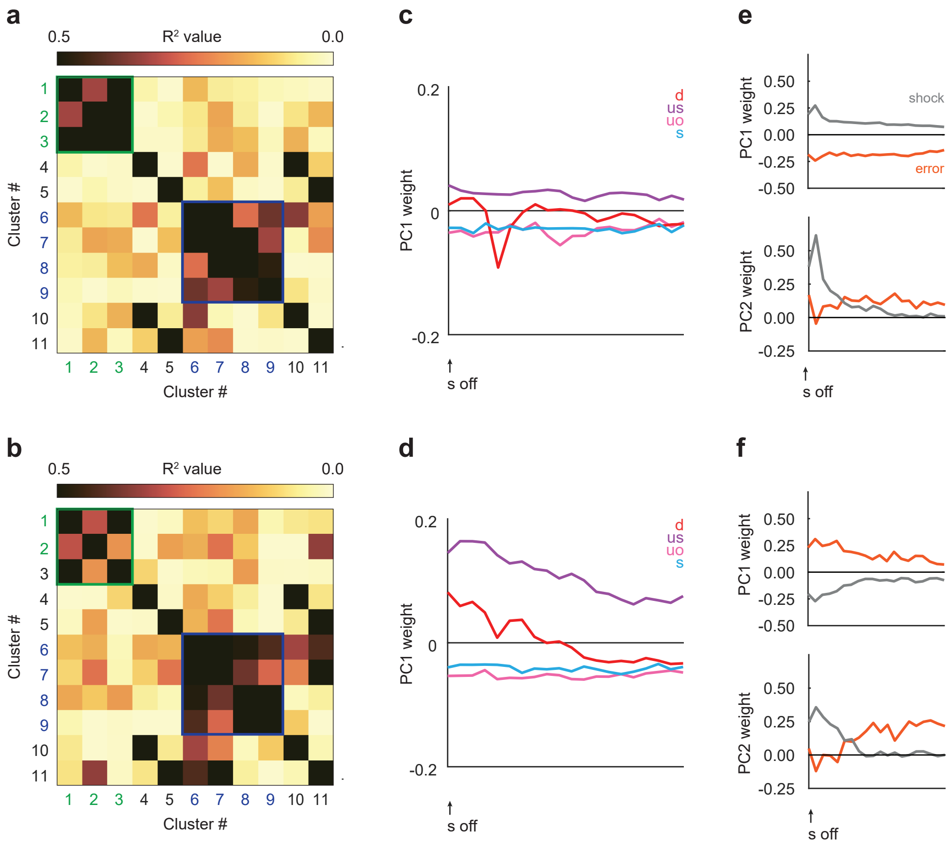

Supplemental Fig. 7 **Brainstem outcome firing split by sex**. Between cluster firing correlations for units from female (a) and male (b) rats. PC1 for brainstem outcome firing, for units from female (c) and male rats (d). PC1 and PC2 resulting from principal components for cluster beta weights for units from female rats (e) and male rats (f). Abbreviations: d, danger; us, uncertainty shock; uo, uncertainty omission, and s, safety.

Supplementary Table 1. **Recording summary.** For each subject: sex, age in days at the start of recording, the number of recording sessions completed, the reason recording was terminated, how many times the probe had been used, and if it was successfully explanted.

Subject	Sex	Age (days)	Sessions	Recording termination	Probe use #	Explanted
1	F	131	8	Reached 8 sessions	1	Yes
2	F	124	9	Reached 9 sessions	2	Yes
3	F	127	10	Reached 10 sessions	1	Yes
4	M	99	2	Lost signal	1	Yes
5	M	95	8	Reached 8 sessions	1	Yes
6	M	115	1	Lost signal	1	Yes
7	M	115	10	Reached 10 sessions	1	Yes
8	M	116	7	broken headcap	1	Yes
9	M	117	10	Reached 10 sessions	1	Yes
10	F	140	10	Reached 10 sessions	1	Yes

Supplementary Table 2. **Number of units per region, per subject.** For each brain region, organized dorsal to ventral, the total number of units recorded from each subject, the total overall units recorded from each subject, and the mean number of accepted units recorded per session for each subject.

Subject	1	2	3	4	5	6	7	8	9	10
DPG	13			7					112	13
DPwh	19			2		16	1		9	25
DLPAG	61	9					28		33	48
LPAG	12	7	29	2	14			43		15
VLPAG	12	5	24	2	22	1	7	18	26	30
Me5						2				
DRL				1						
DRD	5	55							28	11
DRV	17	41							150	
PDR	7			3						17
mlf	11	9								37
isRt						5	28	17		
Pa4						3	1	17		
x/scp	11			2				21		
SPTg			50	7		11	4			
PNO			38			5	23			35
VTg				6						29
DMTg					5					
ts							24	79		
PMnR		130			11				51	44
MnR	24	72								
Total	192	328	141	32	52	43	116	195	409	304
# units per session	24	36.44444	14.1	16	6.5	43	11.6	27.85714	40.9	30.4

Supplementary References

1. Siegle, J., Kulik, P., & Doshi, A. Neuropixels PXI plugin. https://github.com/open-ephys-plugins/neuropixels-pxi (2019).
2. Kulik, P. NI-DAQ plugin. https://github.com/open-ephys-plugins/nidaq-plugin (2019).
3. Steinmetz, N. spikes. https://github.com/cortex-lab/spikes/ (2019).
4. Paxinos, G. and Watson, C. (2007) The Rat Brain in Stereotaxic Coordinates. 6th Edition, Academic Press, San Diego.
5. F. Crameri, Scientific colour maps (Version 4.0.0). (2018), doi:10.5281/zenodo.2649252.
