# Supplementary material for: Brainstem networks construct threat probability and prediction error from neuronal building blocks
Source: Nat Commun. 2022 Oct 19;13:6192. doi: 10.1038/s41467-022-34021-1 (PMC9582012; doi:10.1038/s41467-022-34021-1)
Supplement: Supplementary file 3 — Reporting Summary [file 41467_2022_34021_MOESM3_ESM.pdf]

nature portfolio

Corresponding author(s): Jasmin A Strickland
Michael A McDannald

Last updated by author(s): Dec 3, 2021

Reporting Summary

Nature Portfolio wishes to improve the reproducibility of the work that we publish. This form provides structure for consistency and transparency in reporting. For further information on Nature Portfolio policies, see our Editorial Policies and the Editorial Policy Checklist.

Statistics

For all statistical analyses, confirm that the following items are present in the figure legend, table legend, main text, or Methods section.

n/a	Confirmed	
☐	☒	The exact sample size (*n*) for each experimental group/condition, given as a discrete number and unit of measurement
☐	☒	A statement on whether measurements were taken from distinct samples or whether the same sample was measured repeatedly
☐	☒	The statistical test(s) used AND whether they are one- or two-sided<br>*Only common tests should be described solely by name; describe more complex techniques in the Methods section.*
☒	☐	A description of all covariates tested
☐	☒	A description of any assumptions or corrections, such as tests of normality and adjustment for multiple comparisons
☐	☒	A full description of the statistical parameters including central tendency (e.g. means) or other basic estimates (e.g. regression coefficient) AND variation (e.g. standard deviation) or associated estimates of uncertainty (e.g. confidence intervals)
☐	☒	For null hypothesis testing, the test statistic (e.g. *F*, *t*, *r*) with confidence intervals, effect sizes, degrees of freedom and *P* value noted<br>*Give P values as exact values whenever suitable.*
☒	☐	For Bayesian analysis, information on the choice of priors and Markov chain Monte Carlo settings
☒	☐	For hierarchical and complex designs, identification of the appropriate level for tests and full reporting of outcomes
☐	☒	Estimates of effect sizes (e.g. Cohen's *d*, Pearson's *r*), indicating how they were calculated

Our web collection on statistics for biologists contains articles on many of the points above.

Software and code

Policy information about availability of computer code

Data collection	Open Ephys GUI v0.4.4.1 with Neuropix-PXI plugin v0.3.0, Med-PC V.
Data analysis	Kilosort 2, Kilosort 2.5, Phy 2.0a1, Matlab 2021a

For manuscripts utilizing custom algorithms or software that are central to the research but not yet described in published literature, software must be made available to editors and reviewers. We strongly encourage code deposition in a community repository (e.g. GitHub). See the Nature Portfolio guidelines for submitting code & software for further information.

Data

Policy information about availability of data

All manuscripts must include a data availability statement. This statement should provide the following information, where applicable:
- Accession codes, unique identifiers, or web links for publicly available datasets
- A description of any restrictions on data availability
- For clinical datasets or third party data, please ensure that the statement adheres to our policy

Data will be uploaded and available at https://crcns.org/

Field-specific reporting

Please select the one below that is the best fit for your research. If you are not sure, read the appropriate sections before making your selection.

☒ Life sciences ☐ Behavioural & social sciences ☐ Ecological, evolutionary & environmental sciences

For a reference copy of the document with all sections, see nature.com/documents/nr-reporting-summary-flat.pdf

Life sciences study design

All studies must disclose on these points even when the disclosure is negative.

Sample size	A total of 10 rats were implanted and recorded from. The high data output from each subject and complex data acquisition/analysis justifies this sample size.
Data exclusions	Single unit data were only included for analysis if met a strict set of criteria detailed in the methods section of the manuscript and further in the supplemental methods.
Replication	Study has not yet been replicated in full, however data were collected in two separate rounds of recording from across 10 different subjects, and pooled.
Randomization	Auditory stimuli used in the fear discrimination procedure were counterbalanced taking sex and round of recording into account.
Blinding	All rats underwent the same testing procedure, the nature of the experimental design was such that there were not separate experimental and control groups.

Reporting for specific materials, systems and methods

We require information from authors about some types of materials, experimental systems and methods used in many studies. Here, indicate whether each material, system or method listed is relevant to your study. If you are not sure if a list item applies to your research, read the appropriate section before selecting a response.

Materials & experimental systems

n/a	Involved in the study	
☒	☐	Antibodies
☒	☐	Eukaryotic cell lines
☒	☐	Palaeontology and archaeology
☐	☒	Animals and other organisms
☒	☐	Human research participants
☒	☐	Clinical data
☒	☐	Dual use research of concern

Methods

n/a	Involved in the study	
☒	☐	ChIP-seq
☒	☐	Flow cytometry
☒	☐	MRI-based neuroimaging

Animals and other organisms

Policy information about studies involving animals; ARRIVE guidelines recommended for reporting animal research

Laboratory animals	10 Long Evans rats (6 Male, 4 female)
Wild animals	Study did not involved wild animals
Field-collected samples	Study did not involve samples collected from the field
Ethics oversight	All protocols were approved by the Boston College Animal Care and Use Committee, and all experiments were carried out in accordance with the NIH guidelines regarding the care and use of rats for experimental procedures.

Note that full information on the approval of the study protocol must also be provided in the manuscript.
